# The Effect of Allograft Inflammatory Factor-1 on Inflammation, Oxidative Stress, and Autophagy via miR-34a/ATG4B Pathway in Diabetic Kidney Disease

**DOI:** 10.1155/2022/1668000

**Published:** 2022-10-29

**Authors:** Hao Jianbing, Liu Xiaotian, Tang Jie, Chang Xueying, Jin Honge, Zhu Bo, Hao Lirong, Zhang Lei

**Affiliations:** ^1^Department of Nephropathy and Hemodialysis, Southern University of Science and Technology Hospital, Shenzhen 518055, China; ^2^Department of Neurology, The Third Affiliated Hospital of Heilongjiang University of Chinese Medicine, Harbin 150040, China; ^3^Department of Blood Purification, The First Affiliated Hospital of Harbin Medical University, Harbin 150001, China

## Abstract

Increasing evidence suggests that disorders of inflammation, oxidative stress, and autophagy contribute to the pathogenesis of diabetic kidney disease (DKD). This study attempted to clarify the effect of allograft inflammatory factor-1 (AIF-1), miR-34a, and ATG4B on inflammation, oxidative stress, and autophagy in DKD both *in vitro and in vivo* experiments. *In vivo*, it was found that the levels of AIF-1, miR-34a, oxidative stress, and inflammatory factors were significantly increased in blood and urine samples of DKD patients and mouse models and correlated with the level of urinary protein. *In vitro*, it was also found that the expressions of AIF-1, miR-34a, ROS, and inflammatory factors were increased, while ATG4B and other autophagy related proteins were decreased in human renal glomerular endothelial cells (HRGECs) cultured with high concentration glucose medium (30 mmol/L). When AIF-1 gene was overexpressed, the levels of miR-34a, ROS, and inflammatory factors were significantly upregulated, and autophagy-related proteins such as ATG4B were downregulated, while downregulation of AIF-1 gene had the opposite effect. In addition, miR-34a inhibited the expression of ATG4B and autophagy-related proteins and increased the levels of ROS and inflammation. Furthermore, the result of luciferase reporter assay suggested that ATG4B was the target gene of miR-34a. When ATG4B gene was overexpressed, the level of autophagy was upregulated, and inflammatory factors were downregulated. Conversely, when ATG4B gene was inhibited, the level of autophagy was downregulated, and inflammatory factors were upregulated. Then, autophagy inducers inhibited the levels of inflammation and ROS, whereas autophagy inhibitors had the opposite function in HRGECs induced by glucose (30 mmol/L). In conclusion, the above data suggested that AIF-1 regulated the levels of inflammation, oxidative stress, and autophagy in HRGECs via miR-34a/ATG4B pathway to contribute to the pathogenesis of diabetic kidney disease.

## 1. Introduction

At present, diabetic kidney disease (DKD) as a serious complication of diabetes mellitus has become a leading cause of chronic renal failure on a global level [1–5]. It is very well recognized that excessive inflammation and oxidative stress in diabetes are an important cause of DKD [6–10]. Under physiological state, oxidative stress and inflammation are important to maintaining vital functions, but excessive oxidative stress and inflammation might cause diseases such as DKD. Increasing evidence suggests that treatment with antioxidative or anti-inflammatory drugs could inhibit the progress of DKD [11–13]. For example, SGLT2i or GLP-1R agonists attenuate inflammation and oxidative stress to contribute to reduce urinary protein in DKD patients [11]. In addition, it has been suggested that there is an interaction between inflammation and oxidative stress. Oxidative stress is activated by inflammation, and inflammation is also induced by oxidative stress. When the level of oxidative stress is increased, the expression of chemokines and cytokines which increase inflammation is also upregulated [14]. Moreover, autophagy as an essential process for normal cell homeostasis is considered to defend against oxidative stress and inflammation [15]. It has been reported that autophagy regulates the level of oxidative stress and inflammation in kidney injury [16]. However, the exact mechanisms still need to be fully elucidated.

Autophagy as a self-protection response to inflammation and oxidative stress protects renal cells against injury in DKD, including podocytes, proximal tubular, mesangial, and endothelial cells. On the one hand, intact autophagic flux contributes to maintaining podocyte homeostasis. Dysfunction of autophagy in the podocyte is responsible for the progression of DKD [17–19]. On the other hand, high concentration glucose inhibits autophagy of mesangial cells by upregulating p62/SQSTMI and downregulating LC3 expression [20, 21]. Xu et al. also have confirmed that autophagy contributes to the survival of mesangial cells [22]. In addition, it has been confirmed that autophagy in renal tubular epithelial cells has a protective function in DKD [23]. Based on the reports mentioned above, although increasing evidence has suggested that dysregulated autophagy in glomerular and tubular cells is strongly associated with the pathogenesis of DKD, the exact mechanism of autophagy remains to be elucidated. An increasing number of studies have shown that there is an interplay between inflammation, oxidative stress, and autophagy which play an important role in DKD [24, 25]. Autophagy as an evolutionarily conserved cellular process protects renal cells from damage by inhibiting oxidative stress and inflammation in DKD [26, 27]. It is reported that the oxidative stress in glomerular endothelial cells induced by sustained high concentration blood glucose is a key link for DKD [28]. What is more, autophagy of glomerular endothelial cells protects glomeruli from oxidative stress and maintains the integrity of glomerular capillaries [29]. Enhancing endothelial autophagy may provide a novel therapeutic approach to improving glomerular diseases [30–32]. Therefore, regulation of autophagy in glomerular endothelial cells is helpful for the prevention of DKD. However, how to maintain autophagy of glomerular endothelial cells at the appropriate level is still unclear.

MicroRNAs have a comprehensive regulatory effect, such as cell growth and apoptosis, blood cell differentiation, and homeobox gene regulation, which brings hope for accurate regulation of autophagy [33]. Wang et al. confirmed that many microRNAs, especially miR-34a, were significantly increased in serum and kidney of early DKD mice [34], but the biological functions in DKD are not clear. In our previous study on the role of miR-34a in chronic kidney disease-related vascular calcification [35], we found that autophagy-related gene 4B (ATG4B) was not only a potential target gene for miR-34a regulation but also a key gene for autophagy regulation. Liu et al. suggest that miR-34a via ATG4B regulates the level of autophagy in renal tubular epithelial cells in a mouse model of acute kidney injury [36]. However, whether miR-34a/ATG4B has the similar role in glomerular endothelial cells is not reported. In addition, our previous study has confirmed that AIF-1 could induce smooth muscle cell calcification and the expression of miR-34a in inflammation [37]. AIF-1 plays an important role in many chronic diseases, especially inflammatory responses, and DKD is also known as a chronic inflammatory disease. Our latest research shows that AIF-1 contributes to the pathogenesis of DKD [38]. However, the exact mechanism of AIF-1 in DKD remains unclear.

**Figure 1:**
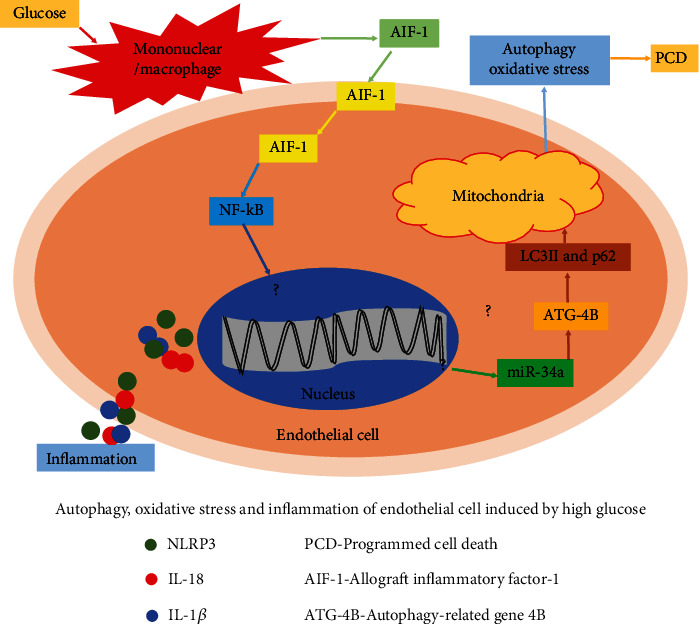
Schematic diagram of possible mechanism of AIF-1 in oxidative stress, inflammation, and autophagy of glomerular endothelial cell induced by glucose.

Therefore, based on the previous studies, it is proposed that there might be interaction between AIF-1, miR-34a, and ATG4B to induce inflammation, oxidative stress, and autophagy in DKD (Figure 1). The study intends to explore the interactions of AIF-1, miR-34a, and ATG4B on inflammation, oxidative stress, and autophagy via *in vivo* and *in vitro* experiments.

## 2. Materials and Methods

### 2.1. Patients

In accordance with the clinical study program approved by the ethics committee of Southern University of Science and Technology Hospital and the ethical standards of the 1964 Helsinki Declaration, we randomly collected 5 mL samples of blood and urine from 60 diabetic kidney diseases (ACR: urinary albumin to creatinine ratio; group 1, 30 mg/g ≤ ACR ≤ 300 mg/g; group 2, ACR > 300 mg/g) and 30 diabetic patients (ACR < 30 mg/g). 30 healthy people without any underlying disease served as the control group. Then, the collected samples were centrifuged and used for further biochemical tests. All patients signed consent for the use of urine and serum specimens.

### 2.2. Animal Model

The *db/db* mice (4-week-old males and females are equally divided) as a spontaneous type 2 diabetic model were divided into five groups by random number table method as follows: model control group (no intervention), miR-34a agonist group (miR-34a agonist injected into caudal vein at an initial dose of 20 nmol and a maintenance dose of 5 nmol every 3 days), miR-34a agonist control group (negative control of miR-34a agonist was intravenously injected by caudal vein), miR-34a antagonist group (miR-34a antagonist was injected by caudal vein at an initial dose of 200 nmol and maintenance dose of 50 nmol every 3 days), and miR-34a antagonist control group (negative control of miR-34a antagonist was injected intravenously by caudal vein at the same dose of antagonist). The *db/m* mice were used as the control group. At the same time, to elucidate the effect of AIF-1 on DKD, another type 2 diabetic kidney disease model was established with *AIF-1* transgenic mice which treated with unilateral renal artery ligation + low − dose intraperitoneal injection of streptozotocin (25 mg/kg, every two days for a week) + high − calorie diet [39, 40]. All mice were grouped according to the above method, when blood glucose > 16.5 mmol/L and urinary albumin/creatinine > 30 mg/g were a successful DKD model. At the end of the experiment, when urine samples were collected for 24 hours, all mice were anesthesia (pentobarbital sodium; 150 mg/kg) by intraperitoneal injection and euthanized. The kidney and blood specimens were collected from each mouse as soon as possible. After centrifugation, the serum was stored at -80°C for later use. The kidney samples were fixed in 4% paraformaldehyde for 24 h at room temperature or stored at -80°C after further study. The whole animal experiments were carried out according to research protocols approved by the Animal Ethics Review Committee of Harbin Medical University or Southern University of Science and Technology and adhered to the principles stated in the Guide for the Care and Use of Laboratory Animals published by the US National Institutes of Health.

### 2.3. Cell Culture and Transfection

Human renal glomerular endothelial cells (HRGEC, USA) were purchased from ScienCell Research Laboratories Company. Cells were cultured with endothelial cell medium (ECM, ScienCell, USA) supplemented with 10% fetal bovine serum for 2-3 days and cultured with serum-free ECM when cells were fused up to 80% for 24 hours for standby use. Then, the cells received the following treatment: normal glucose (5.6 mmol/L glucose), high concentration glucose (10, 15, 20, and 30 mmol/L), miR-34a mimic, miR-34a inhibitor, rapamycin (autophagy inducer), and DC661 (autophagy inhibitor).

According to the manufacturer's protocol, miR-34a mimic (50 nmol) and miR-34a inhibitor (100 nmol, RiboBio, China) were transiently transfected into HRGEC using riboFECT™ CP transfection agent and collected after culture for 48 hours. Incubation with the transfection reagent alone served as a control.

### 2.4. qPCR Analysis (SYBR Method)

Total miRNAs from clinic specimens or cultured HRGEC were extracted using the total miRNAs Isolation Kit (Qiagen, Germany), according to the manufacturer's protocols. Templates for miR-34a were prepared with the miScript II RT Kit (Qiagen, Germany). Bulge-Loop primers of miR-34a (RiboBio, China) and SYBR Green Assays (TaKaRa, Japan) were used for qPCR in a Roche LightCycler480 II. U6 was used as an endogenous reference gene for normalization of the data. qPCR was also performed using SYBR Green Assays. The relative expression level was determined by LightCycler480 Software 1.5, as described by the manufacturer.

### 2.5. Plasmid Construct and Transfection

AIF-1 CRISPR Activation Plasmid (h, sc-400513-ACT), AIF-1 siRNA (h, sc-43857) Atg4b Lentiviral Activation Particles (h, sc-404173-LAC), Atg4b siRNA (h, sc-72584), and control siRNA-A (sc-37007) were purchased from Santa Cruz Biotechnology, Inc. According to manufacturer's instructions, all plasmids were transfected into HRGEC using riboFECT™ CP (RiboBio, Guangzhou, China) as the transfection agent. All plasmids were sequenced to ensure authenticity.

### 2.6. Luciferase Reporter Assay

To construct a luciferase reporter containing wild-type ATG4B-3′UTR (pGL3-ATG4B-WT), a segment of human ATG4B was amplified by PCR using the following primers: Fwd: 5′- CCGCTCGAGGGCGCCGGCCGGATCGATCG-3′ Rev: 5′- CCCAAGCTTCCATCTTGC- GGTACGGACGT-3′ [41] from human genomic DNA and inserted into the pGL3 basic luciferase reporter vector (Promega, Madison, USA). Three-point mutations in the 3′UTR region of pGL3-ATG4B-WT were induced using a Quick Change site-directed mutagenesis kit (Strata-gene, La Jolla, CA, USA), resulting in mutant pGL3-ATG4B (pGL3-ATG4B-MUT). In addition, plasmid DNA was sequenced for authenticity. The luciferase reporters and miR-34a mimics or miRNA controls were cotransfected into HRGEC. Forty-eight hours after transfection, luciferase activities were detected using the Luciferase Assay System (Promega Biotech, USA).

### 2.7. Immunohistochemical Staining for Histological Analysis

Specimens of kidney in experimental mice were fixed with 10% buffered formalin and embedded in paraffin, and 4-micron tissue sections were prepared and treatment as our previous study [37]. Then, the tissue sections were incubated with primary antibodies against AIF-1 (1 : 1,000, Abcam, UK) at 4°C overnight. After rinsing with PBS, the tissue sections were incubated with goat antimouse antibody at 37°C for 1 h. Finally, the immunocomplexes were stained with DAB and observed under a microscope (Nikon Corp., Japan).

### 2.8. Western Blot Analyses

Protein concentrations were determined using the Bradford protein assay. For electrophoresis, 50 *μ*g of total protein was loaded onto 10% SDS-PAGE gels using a Bio-Rad Mini Gel apparatus. After electrophoresis, the separated proteins were transferred onto a polyvinylidene difluoride membrane (Millipore, Billerica, MA, USA). The membrane was blocked in western blocking buffer for 1 h at room temperature and then incubated with primary antibodies against AIF-1, ATG4B (1 : 2000, Abcam), p62, LC3B, and *β*-actin (1 : 1000; Santa Cruz) for 2 h. Membranes were then incubated with goat antimouse/goat antirabbit secondary antibodies (1 : 10000; DyLight®800, Immuno Reagents, USA) for 1 h. The bands in the membrane were visualized and analyzed using the Odyssey Imaging System (LICOR Bioscience, USA). Protein levels were quantified and normalized to *β*-actin levels.

### 2.9. Reactive Oxygen Species (ROS) Assay

The intracellular ROS in HRGEC was detected by ROS assay kit (Beyotime Biotechnology Co., Ltd.) according to the manufacturer's protocols. Briefly, DCFH-DA (2,7-dichlorodi-hydrofluorescein diacetate) were diluted in serum-free medium at 1 : 1000 to a final concentration of 10 mmol/L. The medium was discarded and added DCFH-DA diluted in appropriate volume at 37°C for 20 minutes. The cells were washed three times with serum-free culture medium to fully remove the residual DCFH-DA. The fluorescence was read at 485 nm for excitation and 530 nm for emission with a fluorescence plate reader (Genios, TECAN). ROS level was quantified by measurement of fluorescence intensity.

### 2.10. ELISA Analysis

The levels of AIF-1, ATG4B, NLRP3, IL-1*β*, and IL-18 in serum or urine were detected by human or mouse ELISA kit (Shanghai Jianglai Biotechnology Co., Ltd., China), according to the manufacturer's instructions. In simple terms, 50 *μ*L sample of serum, urine, or cell supernatant was added into the enzyme label plate and incubated at 37°C for 30 min. After washing the plate 5 times, 50 *μ*L enzyme standard reagent was added and incubated at 37°C for 30 min. After washing the plate 5 times, 50 *μ*L color solution A or B was added and incubated at 37°C for 15 min. 50 *μ*L stop solution was added and read by semiautomatic single channel microplate reader (Thermo Multiskan FC, USA) at 450 nm immediately. The software of Curve Expert 1.30 was used to draw the standard curve. According to the OD value of the standard sample, the regression equation was calculated from the standard curve. Then, the OD value of the sample was substituted into the equation to calculate the concentration of sample.

### 2.11. Physiological Indicator Measurements

Physiological and biochemical indicators of all volunteers were collected from medical records. The levels of physiological and biochemical indicators in experimental mice were measured commercial assay kits. Basically, fasting plasma glucose (FPG) was measured by a glucose oxidase method using a glucose analyzer. Plasma insulin was measured using an automated chemiluminescence system (ADVIA Centaur Immunoassay System, Siemens Healthcare Diagnostics, USA). Blood lipids, nitrogen (BUN), and serum creatinine (Scr) were measured by commercial assay kits (Roche Diagnostics, Basel, Switzerland) on an automatic blood chemistry analyzer (Roche-Hitachi 7180, Roche Diagnostics, Basel, Switzerland). Estimated glomerular infiltration rate (eGFR) was calculated using the 2009 Chronic Kidney Disease Epidemiology Collaboration Equation. UACR was measured by immunonephelometric assay method.

### 2.12. Statistical Analysis

All experimental results were presented as mean ± standard deviation. Analysis of variance together with the least significant difference post hoc test was used to assess differences between multiple groups using SPSS25.0 for Windows (SPSS, USA). All experiments were repeated for 3 times, and *P* < 0.05 was considered statistically significant.

## 3. Results

### 3.1. The Levels of Physiological Indicators and Inflammatory Factors in Serum and Urine of DKD Patients

It is well accepted that diabetes is a metabolic disease with many abnormal physiological indicators and excessive inflammation. To clarify the general condition of the enrolled patients, assay or qPCR was used to measure the physiological indicators and inflammatory factors of serum or urine. As the results showed in Table 1, physiological indicators such as blood glucose, islet function, blood lipids, kidney function, and urinary protein were abnormal, except liver function, compared with healthy people. Furthermore, the levels of AIF-1, miR-34a, NLRP3, IL-1*β*, and IL-18 in serum or urine were increased except ATG4B protein (Tables 1 and 2, *P* < 0.01). The data mentioned above showed that the levels of inflammatory factors were increased, and autophagy-related protein was decreased in DKD patients with albuminuria. It suggested that inflammation and autophagy were involved in DKD.

### 3.2. The Levels of Autophagy and Inflammation in *db/db* Mice

**Figure 2:**
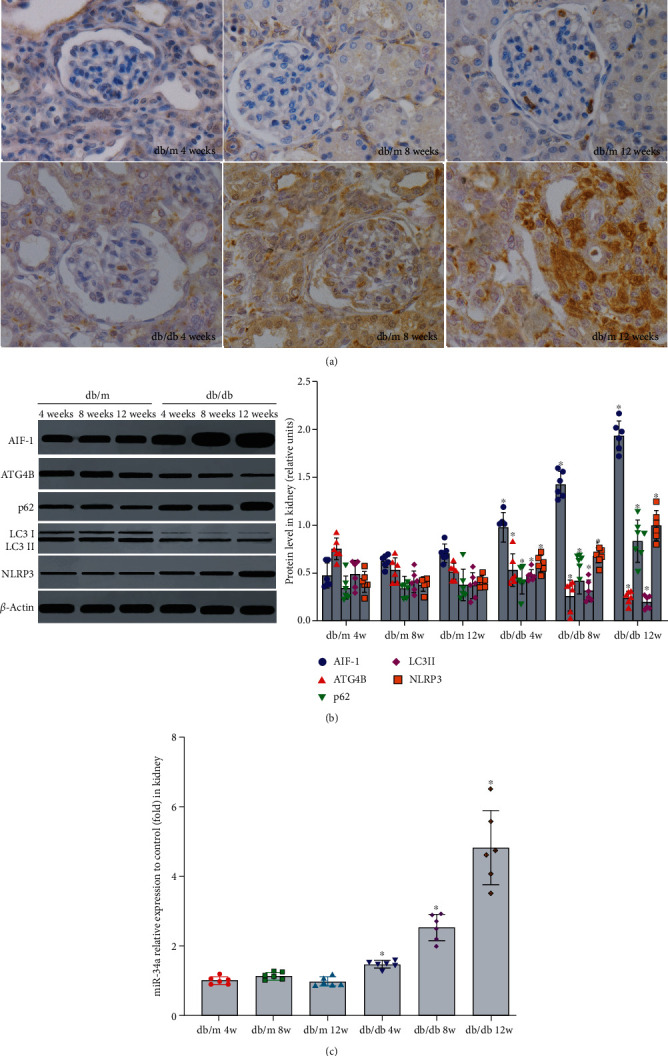
The levels of AIF-1, miR-34a, ATG4B, autophagy, and inflammation in kidney of *db/db* mice. (a) The expression of AIF-1 in *db/db* mice was evaluated by immunohistochemical staining. (b) The expression of AIF-1, ATG4B, LC3II, p62, and NLRP3 protein in *db/db* mice was detected by western blot. Versus *db/m* mice group, ^∗^*P* < 0.01. (c) The expression of miR-34a in *db/db* mice was detected by qPCR. Versus *db/db* mice group, ^∗^*P* < 0.001. AIF-1: allograft inflammatory factor-1; miR-34a: microRNA 34a; ATG4B: autophagy related 4 homolog B; LC3II and p62: autophagy-associated protein; NLRP3: NOD-like receptor thermal protein domain associated protein 3.

To determine the levels of autophagy and inflammation in DKD model, 4-week-old *db/db* mice which were spontaneous type 2 diabetic models and were fed with high-calorie diet for 4, 8, and 12 weeks. Compared with the control group (*db/m* mice), blood glucose, blood lipids, urinary albumin, AIF-1, miR-34a, NLRP3, IL-1*β*, and IL-18 in serum or urine were increased in *db/db* mice (Table 3, *P* < 0.01). In addition, the expression of AIF-1, miR-34a, p62, and NLRP3 in the kidney was significantly upregulated, whereas the expression of ATG4B and LC3II was significantly downregulated (Figure 2, *P* < 0.01). The above data showed that the level of autophagy was decreased, and the level of inflammation was increased in *db/db* mice.

### 3.3. The Effect of miR-34a on Autophagy and Inflammation in *db/db* Mice

**Figure 3:**
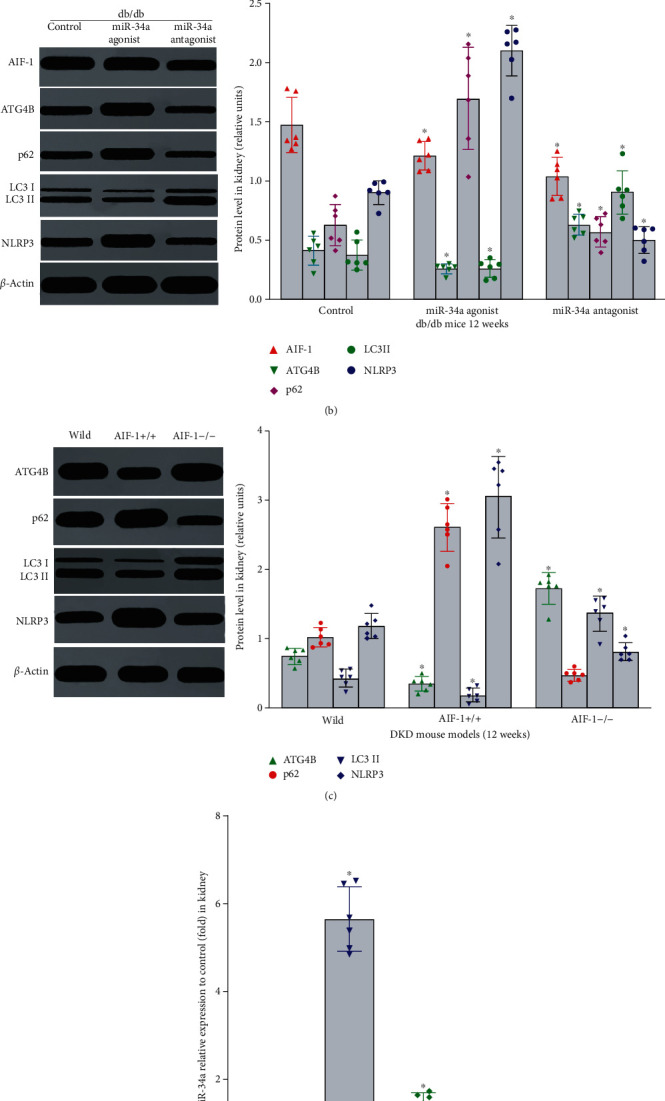
The effect of AIF-1 or miR-34a on autophagy and inflammation in DKD mice. (a) The expression of AIF-1 in *db/db* mice treatment with miR-34a agonist or antagonist was evaluated by immunohistochemical staining. (b)The effect of miR-34a on the expression of AIF-1, ATG4B, LC3II, p62, and NLRP3 protein in *db/db* mice was detected by western blot. Versus *db/m* mouse group, ^∗^*P* < 0.01. (c) The expression of ATG4B, LC3II, p62, and NLRP3 protein in *AIF-1* transgenic mice was detected by western blot. Versus wild mice group, ^∗^*P* < 0.001. (d) The expression of miR-34a in *AIF-1* transgenic mice was detected by qPCR. Versus wild mice group, ^∗^*P* < 0.001. AIF-1: allograft inflammatory factor-1; miR-34a: microRNA 34a; ATG4B: autophagy related 4 homolog B; LC3II and p62: autophagy-associated protein; NLRP3: NOD-like receptor thermal protein domain associated protein 3.

It has been confirmed that many microRNAs such as miR-34a are significantly increased in serum and kidney of DKD patients and play an important role in regulating biological functions. To investigate the effects of miR-34a on autophagy and inflammation in DKD, miR-34a agonist or antagonist was used to regulate the level of miR-34a in *db/db* mice. Compared with the control group, treatment with miR-34a agonist reduced urinary albumin and miR-34a antagonist induced urinary albumin. At the same time, the expression of p62, NLRP3, IL-1*β*, and IL-18 was increased, whereas ATG4B and LC3II were decreased in miR-34a agonist group (Table 4, Figure 3(b), *P* < 0.01). However, miR-34a antagonist group had the opposite effect in Figure 3(b) (*P* < 0.01). In addition, the results mentioned in Figures 3(a) and 3(b) suggested that there was no significant difference in AIF-1 expression between the miR-34a agonist and inhibitor groups (*P* > 0.05). Therefore, miR-34a could aggravate urinary albumin in DKD by regulating the level of ATG4B, LC3IIp62, NLRP3, IL-1*β*, and IL-18 directly, except AIF-1.

### 3.4. The Level of Autophagy and Inflammation in *AIF-1* Transgenic DKD Mice

To clarify the role of AIF-1 on autophagy and inflammation in DKD, AIF-1 transgenic mice were used to establish DKD models. Compared with wild mice, the levels of urinary albumin, inflammation, and autophagy were significantly increased in AIF-1 overexpression mice (*AIF-1^+/+^*) and decreased in AIF-1 knock-down mice (*AIF-1^−/−^*). The results showed that the expressions of miR-34a, p62, and NLRP3 were significantly upregulated in *AIF-1^+/+^*mice and downregulated in *AIF-1^−/−^* (Table 5 and Figures 3(c) and 3(d), *P* < 0.01). The above data showed that AIF-1 was involved in DKD via autophagy and inflammation pathway.

### 3.5. The Level of Inflammation, Oxidative Stress, and Autophagy of HRGECs Exposed to High Concentration Glucose

**Figure 4:**
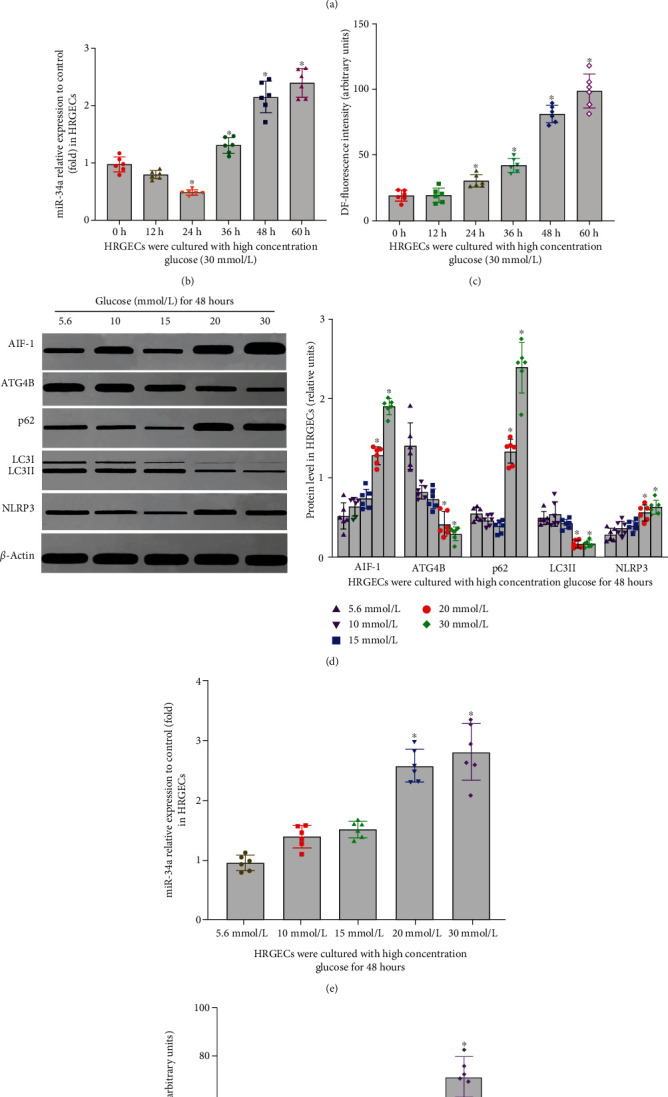
The effect of glucose on autophagy, inflammation, and ROS of HRGECs with a time- and concentration-dependent manner. (a–c) The effect of high concentration glucose (30 mmol/L) on AIF-1, miR-34a, ATG4B, LC3II, p62, NLRP3, and ROS level in HRGECs with a time-dependent manner. Versus 0 h group, ^∗^*P* < 0.001. (d–f) The effect of glucose on AIF-1, miR-34a, ATG4B, LC3II, p62, NLRP3, and ROS level in HRGECs with a concentration-dependent manner for 48 h. All the relative levels of results were corrected by total protein. Versus 5.6 mmol/L glucose group, ^∗^*P* < 0.01. HRGECs: human renal glomerular endothelial cells; AIF-1: allograft inflammatory factor-1; miR-34a: microRNA 34a; ATG4B: autophagy related 4 homolog B; LC3II and p62: autophagy-associated protein; NLRP3: NOD-like receptor thermal protein domain associated protein 3; ROS: reactive oxygen species.

Inflammation, oxidative stress, and autophagy in glomerular endothelial cells play an important role in DKD. To clarify the effect of inflammation, oxidative stress, and autophagy in HRGECs, cells were cultured with different concentration glucose (5.6, 10, 15, 20, and 30 mmol/L) for 0 h, 12 h, 24 h, 36 h, 48 h and 60 h. As the results mentioned in Figure 4, glucose induced the expression of AIF-1, miR-34a, p62, NLRP3, and ROS and inhibited the expression of ATG4B and LC3II in HRGECs with a dose- and time-dependent manner (Figure 4, *P* < 0.01). These results suggested that high concentration glucose regulated the levels of AIF-1, miR-34a, inflammation, oxidative stress, and autophagy in HRGECs.

### 3.6. The Effect of AIF-1 on the Expression of miR-34a and ATG4B in HRGECs Exposed to High Concentration Glucose

**Figure 5:**
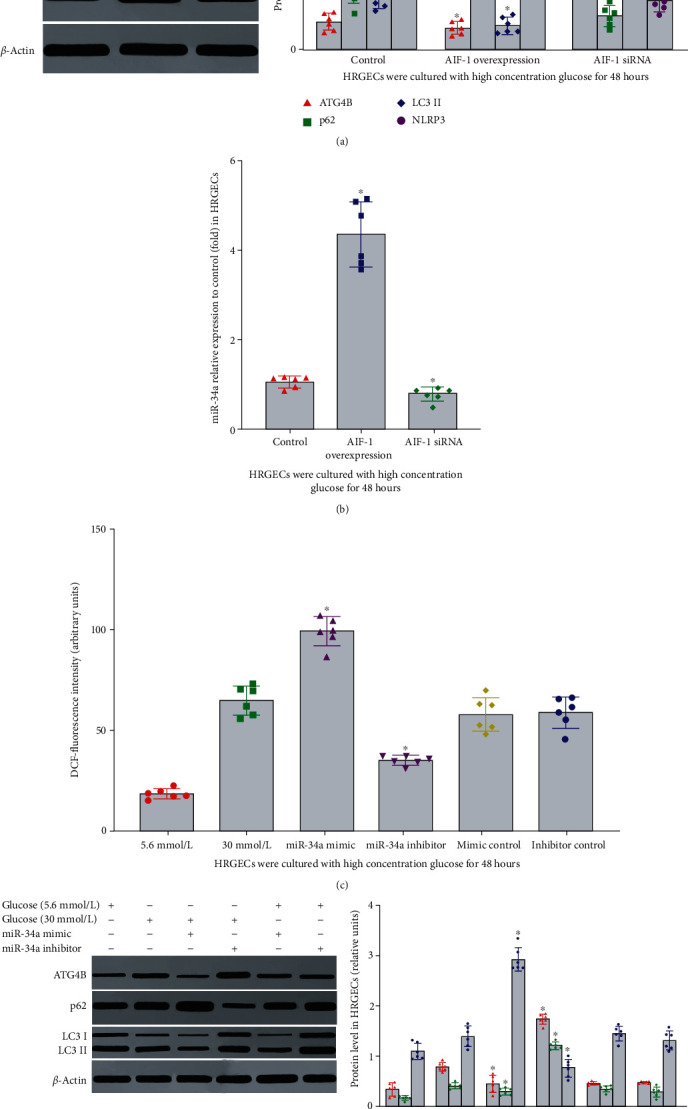
The effect of AIF-1 or miR-34a on autophagy, inflammation, and ROS in HRGECs. (a, b) The effect of AIF-1 on the expression of miR-34a, ATG4B, LC3II, p62, and NLRP3 protein in HRGECs. Versus control group, ^∗^*P* < 0.001. (c, d) The effect of miR-34a on the expression of ATG4B, LC3II, p62, NLRP3, and ROS in HRGECs. All the relative levels of results were corrected by total protein. Versus control group, ^∗^*P* < 0.001. HRGECs: human renal glomerular endothelial cells; AIF-1: allograft inflammatory factor-1; miR-34a: microRNA 34a; ATG4B: autophagy related 4 homolog B; LC3II and p62: autophagy-associated protein; NLRP3: NOD-like receptor thermal protein domain associated protein 3; ROS: reactive oxygen species.

Allograft inflammatory factor 1 (AIF-1) is a highly conserved immunomodulatory inflammatory response calcium-binding protein and involved in various inflammatory reactions. The above data showed that the expression of AIF-1 was significantly upregulated and not affected by miR-34a in *db/db* mice and HRGECs. However, the expression of miR-34a was upregulated in AIF-1 overexpressing mice. To explore the exact effect of AIF-1 on miR-34a and ATG4B, AIF-1 plasmid or siRNA was transfected into HRGECs. When AIF-1 was overexpressed, the levels of miR-34a, p62, NLRP3, and ROS were increased, whereas the levels of ATG4B and LC3II were decreased in HRGECs exposed to high concentration glucose (Figures 5(a) and 5(b), *P* < 0.01). However, AIF-1 siRNA inhibited the expression of miR-34a, p62, NLRP3, and ROS and induced the expression of ATG4B and LC3II (Figures 5(a) and 5(b), *P* < 0.01). Therefore, the results mentioned above have shown that AIF-1 could induce autophagy and inflammation via miR-34a and ATG4B pathway.

### 3.7. The Effect of miR-34a on Inflammation, Oxidative Stress, and Autophagy of HRGECs Exposed to High Concentration Glucose

Because miR-34a has a variety of biological functions, to further clarify the role of miR-34a in autophagy and inflammation induced by high concentration glucose, miR-34a mimics or inhibitors were transfected into HRGECs. Compared with the control group, the expression of p62, NLRP3, and ROS was upregulated, and ATG4B and LC3II were downregulated in miR-34a mimic group (Figures 5(c) and 5(d), *P* < 0.01). In addition, miR-34a inhibitors had the opposite function. However, miR-34a had no obvious effect on the expression of AIF-1. The results mentioned above suggested that miR-34a contributed to autophagy and inflammation in HRGECs cultured in high concentration glucose via regulating the expression of ATG4B, p62, LC3II, NLRP3, and ROS.

### 3.8. ATG4B Was the Target Gene of miR-34a

**Figure 6:**
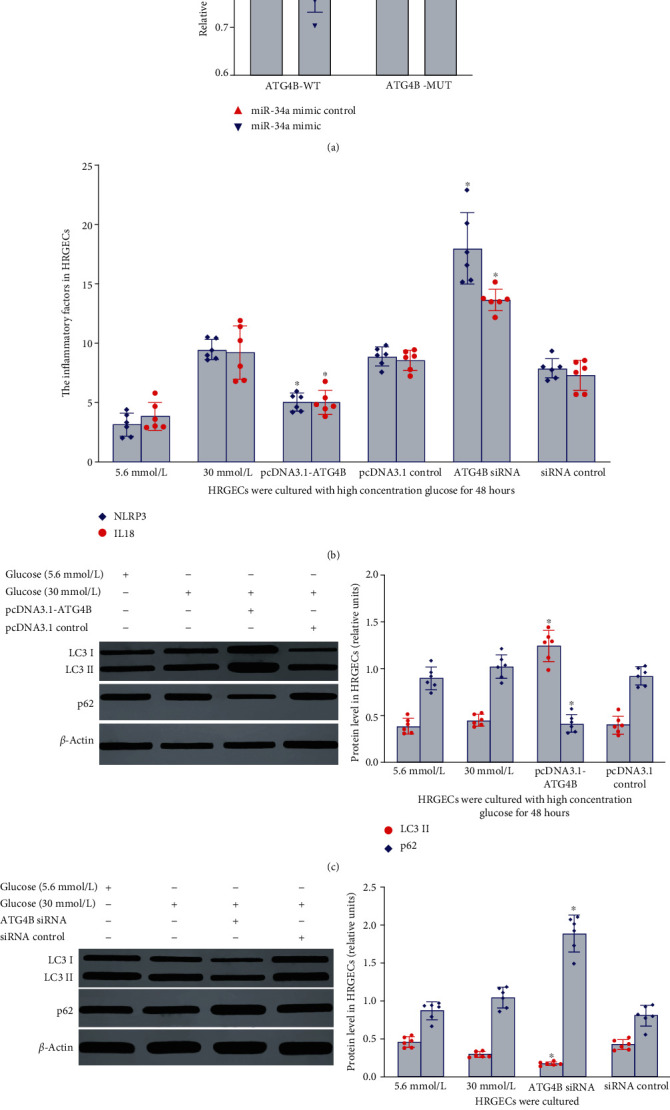
The effect of ATG4B on autophagy and inflammation in HRGECs cultured with high concentration glucose. (a) The relationship between ATG4B and miR-34a was analyzed via luciferase reporter assay. Versus ATG4B-MUT group, ^∗^*P* < 0.05. (b) The effect of ATG4B on the level of NLRP3 and IL18 in HRGECs cultured in 30 mmol/L glucose for 48 h. Versus high glucose control group, ^∗^*P* < 0.01. (c, d) The effect of ATG4B on the expression of LC3II and p62 in HRGECs cultured in 30 mmol/L glucose for 48 h. All the relative levels of results were corrected by total protein. Versus 30 mmol/L glucose control group, ^∗^*P* < 0.001. HRGECs: human renal glomerular endothelial cells; miR-34a: microRNA 34a; ATG4B: autophagy related 4 homolog B; LC3II and p62: autophagy-associated protein; NLRP3: NOD-like receptor thermal protein domain associated protein 3.

The results mentioned above suggested that miR-34a downregulated the expression of ATG4B protein in HRGECs exposed to high concentration glucose. To clarify the relationship between miR-34a and ATG4B, TargetScan.6.2 and http://microRNA.org/ were used to predict the coding sequence of ATG4B as a potential target of miR-34a. Luciferase assay results in Figure 2(d) showed that miR-34a mimic significantly inhibited the luciferase activity of wild-type 3′UTR of the ATG4B reporter gene (Figure 6(a), *P* < 0.05). However, there was no significant repressive effect of the MUT-ATG4B-3-UTR reporter genes (Figure 6(a), *P* < 0.05). The negative control of miRNA simulators had no significant effect on the activity of ATG4B luciferase of wild-type and mutants (Figure 6(a), *P* < 0.05). Furthermore, miR-34a overexpression significantly decreased the expression of ATG4B protein. It was suggested that miR-34a exerted its function via an effect at the posttranscriptional level. Consequently, these results indicated that ATG4B was an important target of miR-34a in HRGECs.

### 3.9. The Effect of ATG4B on Autophagy and Inflammation in HRGECs Exposed to High Concentration Glucose

To further investigate whether ATG4B could regulate HRGECs autophagy, the ATG4B overexpression vector (pcDNA3.1-ATG4B) or ATG4B siRNA was transfected into HRGECs. ATG4B protein overexpression induced the expression of LC3II, whereas inhibited the expression of p62, NLRP3, and IL18 in HRGECs exposed to high concentration glucose (30 mmol/L) for 48 h (*P* < 0.05), as shown in Figures 6(b)–6(d). However, ATG4B siRNA inhibited the expression of LC3II and induced p62, NLRP3, and IL18 expression (*P* < 0.05), as shown in Figures 6(b)–6(d). Control empty vector (pcDNA3.1) and control siRNA did not cause any effects on HRGECs. These results demonstrated that ATG4B participated in autophagy and inflammation of HRGECs induced by high concentration glucose.

### 3.10. The Effects of Autophagy on the Expression of AIF-1, miR-34a, NLRP3, and the Level of ROS in HRGECs Exposed to High Concentration Glucose

**Figure 7:**
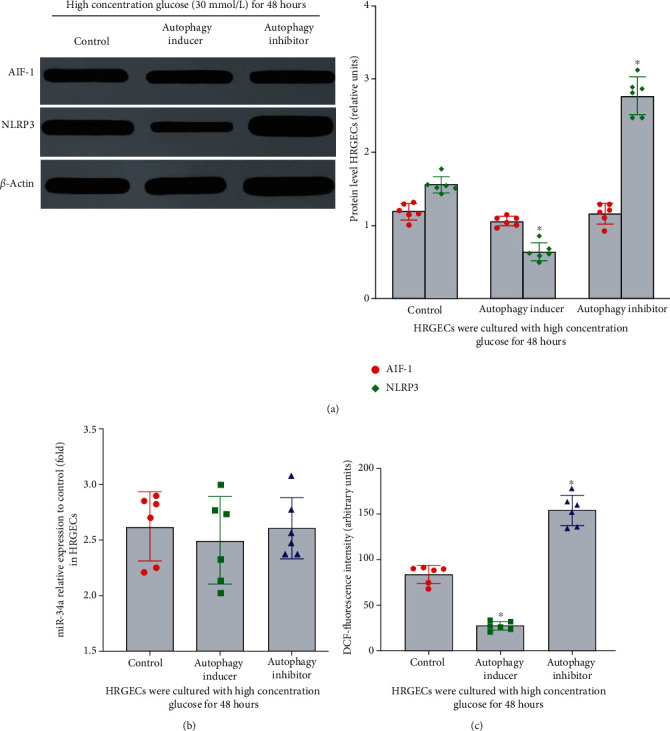
The effect of autophagy on inflammation and ROS in HRGECs cultured with high concentration glucose. (a) The expression of AIF-1 protein was detected by western blot. Versus control group, ^∗^*P* > 0.05. But compared with control group, the expression of NLRP3 protein was upregulated, ^∗^*P* < 0.001. (b) The expression of miR-34a was detected by qPCR. All the relative levels of results were corrected by total protein. Versus control group, ^∗^*P* > 0.05. (c) The effect of autophagy on ROS level in HRGECs cultured in 30 mmol/L glucose for 48 h. Versus high glucose control group, ^∗^*P* < 0.001. HRGECs: human renal glomerular endothelial cells; AIF-1: allograft inflammatory factor-1; miR-34a: microRNA 34a; NLRP3: NOD-like receptor thermal protein domain associated protein 3; ROS: reactive oxygen species.

The results mentioned above suggested AIF-1 and miR-34a inhibited the level of autophagy in HRGECs cultured with high concentration glucose. In contrast, whether autophagy influenced the level of AIF-1, miR-34a, inflammation, and ROS is still unclear. To explore the effect of autophagy on the level of AIF-1, miR-34a, NLRP3, and ROS, HRGECs exposed to high concentration glucose (30 mmol/L) were treated with an autophagy inducers or inhibitors for 48 h. As shown in Figure 7, rapamycin (autophagy inducer) inhibited inflammation and ROS (Figure 7, *P* < 0.001) production, but it did not influence the expression of either AIF-1 or miR-34a (*P* > 0.05). In addition, DC661 (autophagy inhibitor) exerted the opposite effect (Figure 7, *P* < 0.001). Thus, these data suggested that autophagy inhibited the inflammation and ROS of HRGECs induced by high concentration glucose.

## 4. Discussions

Diabetic kidney disease develops in approximately 40% of patients who are diabetic and has become the leading cause of CKD worldwide. Increasing evidence has shown that inflammation, oxidative stress, and autophagy contribute to the pathogenesis of DKD [31, 42], especially the role of autophagy in DKD has been paid increasingly attention all over the world [30, 43–46]. For example, autophagy significantly decreases in the kidney of early stage DKD and is involved in pathogenesis of DKD [47–49]. It might be a new direction of diagnosis and treatment in diabetic nephropathy. However, the exact pathogenesis of DKD and how to maintain the adaptive level of autophagy are still uncertain. It is well known that the glomerulus consists of parietal epithelial cells, podocytes, glomerular endothelial cells (GECs), and mesangial cells. Significantly, GECs cover the luminal surface of glomerular capillaries, expose to circulating high blood glucose levels, and are particularly vulnerable to injury induced by hyperglycemia in the early stage of DKD [50]. Furthermore, the pathogenic signals from GECs are transmitted into podocytes, as well as mesangial cells, and induce a phenotypic switch that modifies their intracellular signaling leading to dysfunction [51–53]. At the present, an increasing number of studies suggest that GECs dysfunction as a key event in the pathogenesis of DKD [54–57]. Thus, it is very important to elucidate the mechanism of GECs dysfunction induced by high glucose for the pathogenesis of diabetic nephropathy. In our previous study, our team has confirmed that AIF-1 facilitates glomerular endothelial cell inflammation and oxidative stress in DKD via the NF-*κ*B signaling pathway [38]. Furthermore, in this study, we demonstrated for the first time that AIF-1 regulated the level of inflammation, oxidative stress, and autophagy in glomerular endothelial cells through miR-34a/ATG4B pathway in diabetic kidney disease.

AIF-1 is an allograft inflammatory factor and plays an important role in regulating the inflammatory response. Our previous studies have confirmed that it is involved in the regulation of inflammatory reactions in various tissues and organs, including peritoneum [58], kidney [59], and blood vessels [37]. Although increasing evidence has confirmed that AIF-1 contributes to the pathogenesis of DKD [38, 60], the exact mechanism is still unclear. In this study, it was demonstrated that AIF-1 regulated the levels of miR-34a, ATG4B, autophagy, inflammation, and oxidative stress in glomerular endothelial cells induced by hyperglycemia via *in vivo* and i*n vitro* studies.

miRNA is a kind of small noncoding RNA molecule (containing about 22 nucleotides) with the function of RNA silencing and posttranscriptional regulation of gene expression [61] which acts both as a functional RNA and a potential biomarker for disease prediction [62]. As a class of small single-stranded noncoding RNA, miR-34a is involved in the pathological process of various diseases such as dietetic kidney disease by regulating the function of target genes such as Egr1, GAS1, and Sirt1/HIF-1*α* [63–67]. Wang et al. have found that miR-34a expression significantly increased in serum and kidney of early DKD mice via gene chips, but the specific mechanism of action is still unclear [34]. In addition, Xiao et al. and Opazo-Ríos et al. also have confirmed that miR-34a contributes to the pathogenesis DKD in rat or mouse models [67–69]. At the present, the mechanism that miR-34a/ATG4B regulated autophagy had already been described in tumor cells [63] and epithelial cells after kidney injury [36]. However, whether there is the similar effect in glomerular endothelia cells has not been investigated. In our study, we found that the levels of inflammation, oxidative stress, and autophagy in HRGECs were regulated via miR-34a/ATG4B pathway in DKD for the first time. In addition, we also clarified that ATG4B was the target gene of miR-34a in HRGECs.

Koch et al. suggest that under normal blood glucose level, autophagy is an important protective mechanism in renal epithelial cells, including podocytes, proximal tubular, mesangial and endothelial cells. However, down regulation of autophagy in hyperglycemic condition, can contribute to the development and progression of diabetic kidney disease [45]. To date, the dysregulation of autophagy is still unclear. It has confirmed that autophagy-related gene (ATG) contributes to the regulation of autophagy, especially ATG4, which is covalently connected with phosphatidylethanolamine (PE) by cutting the c-terminal arginine of ATG8 to form ATG8-PE, which is anchored on the autophagy bubble membrane. Meanwhile, ATG4 removes the fate of ATG8-PE, promotes the fusion of autophagosomes and lysosomes, and induces the formation of autophagosomes [70]. In mammalian cells, ATG4 gene has four subtypes, ATG4A, ATG4B, ATG4C, and ATG4D. Particularly, ATG4B, as the main member of ATG4 family, exists in the cytoplasm and activates ATG8 family (LC3 family and GABARAP), especially LC3B, which activation efficiency is 1500 times of the other three subtypes [71]. Previous studies have identified that the basic level and hunger-induced autophagy significantly decreased in all organizations of ATG4B gene knock-down mice, but the existence of ATG4A/C/D cannot effectively make up for ATG4B lack. On the other hand, the experimental results mentioned above confirmed that there was a positive correlation between ATG4B and autophagy levels in HRGECs cultured with high concentration glucose medium. Therefore, it is conceivable that ATG4B may participate in HRGEC autophagy and oxidative stress.

At present, the main accepted mechanism for DKD is dysregulated autophagy and oxidative stress in glomerular endothelial cells [72–74]. Autophagy protects cells from damage by eliminating damaged proteins and organelles. Clinically, blood and urine biomarkers of autophagic proteins are depressed in the patients with diabetic kidney disease [75, 76], and renal biopsy specimens of patients with insulin resistance exhibit molecular evidence of autophagy suppression [77]. In this study, we found that autophagy was negatively correlated with the level of inflammation and ROS, which was similar to previous research results. Intracellular oxidative stress, which induces autophagy, interacts with autophagy [78], which in turn inhibits oxidative stress and protects cells from damage.

In conclusion, the present study confirmed that AIF-1 regulated the level of autophagy, oxidative stress, and inflammation via miR-34a/ATG4B in HRGECs induced by high concentration glucose. Therefore, regulating autophagy, oxidative stress, and inflammation via AIF-1/miR34a/ATG4B pathway would provide a new therapeutic target for the prevention of DKD.

## Figures and Tables

**Table 1:** Physiological indicators and inflammatory factors in serum of DKD patients.

Clinical parameters	Control (*n* = 30)	Group 1 (*n* = 30)	Group 2 (*n* = 30)	Group 3 (*n* = 30)
FPG (mmol/L)	4.6 ± 1.13	5.4 ± 1.91	4.8 ± 2.38	7.3 ± 2.76^∗^
FINS (*μ*mol/L)	12.3 ± 4.24	21.7 ± 3.87	^∗^25.1 ± 5.72^∗^	22.9 ± 4.65^∗^
Total cholesterol (mmol/L)	4.2 ± 0.61	5.1 ± 0.82	5.6 ± 1.43^∗^	6.1 ± 2.67^∗^
Triglycerides (mmol/L)	1.2 ± 0.47	2.4 ± 0.73^∗^	3.1 ± 1.15^∗^	3.5 ± 1.41^∗^
Serum creatinine (mg/L)	0.8 ± 0.15	1.1 ± 0.26	1.2 ± 0.19^∗^	1.5 ± 0.55^∗^
BUN (mmol/L)	4.5 ± 1.76	5.1 ± 2.18	5.6 ± 2.41	6.8 ± 2.93^∗^
eGFR (ml/min/1.73m^2^)	115.8 ± 20.15	110.1 ± 25.12	95.7 ± 15.63	85.4 ± 12.69^∗^
AST (unit/g)	16.8 ± 3.61	21.5 ± 4.69	19.3 ± 3.62	25.6 ± 5.91
ALT (unit/L)	10.2 ± 2.83	16.7 ± 5.78	26.5 ± 7.43	31.9 ± 6.94
Albumin (g/L)	45.3 ± 4.13	43.3 ± 3.63	41.1 ± 3.46	39.3 ± 3.17
AIF-1 (pg/mL)	50.3 ± 5.23	80.5 ± 7.26^∗^	149.6 ± 8.14^#^	310.1 ± 20.23^#^
miR-34a (fold)	1.0 ± 0.01	1.5 ± 0.12^∗^	2.6 ± 0.23^#^	4.3 ± 0.34^#^
NLRP3 (ng/ml)	2.5 ± 0.23	7.5 ± 1.73^∗^	15.5 ± 2.71^#^	25.8 ± 3.28^#^
ATG4B (ng/ml)	21.2 ± 2.24	19.3 ± 1.58^∗^	13.5 ± 1.22^#^	8.4 ± 2.73^#^
IL-1*β* (pg/mL)	1.5 ± 0.14	3.5 ± 0.41^∗^	9.8 ± 1.91^#^	17.3 ± 3.36^#^
IL-18 (pg/mL)	20.1 ± 3.51	60.2 ± 5.98^∗^	78.2 ± 6.38^#^	96.1 ± 8.53^#^

Control: healthy people; FPG: fasting plasma glucose; FINS: fasting insulin; BUN: urea nitrogen; eGFR: estimated glomerular infiltration rate; AST: aspartate aminotransferase; ALT: alanine transaminase; AIF-1: allograft inflammatory factor-1; miR-34a: microRNA 34a; ATG4B: autophagy related 4 homolog B; NLRP3: NOD-like receptor thermal protein domain associated protein 3; IL-1*β*: interleukin 1*β*; IL-18: interleukin 18; Group 1: 30 mg/g ≤ ACR ≤ 300 mg/g; Group 2: ACR > 300 mg/g; Group 3: normal control group; versus control, ^∗^*P* < 0.01, #*P* < 0.001.

**Table 2:** Clinicopathological factors in urine of DKD patients.

Clinical parameters	Control (*n* = 30)	Group 1 (*n* = 30)	Group 2 (*n* = 30)	Group 3 (*n* = 30)
UACR (mg/L)	11.5 ± 2.45	82.5 ± 20.77^∗^	663.7 ± 119.84^#^	2803.5 ± 864.35^#^
AIF-1 (pg/mL)	40.3 ± 3.23	60.5 ± 5.42^∗^	107.6 ± 9.41^#^	209.1 ± 18.2^#^
miR-34a (fold)	1.1 ± 0.16	1.8 ± 0.21^∗^	3.2 ± 0.35^#^	5.3 ± 0.76^#^
NLRP3 (ng/ml)	0.9 ± 0.14	1.5 ± 0.16^∗^	5.5 ± 0.87^#^	7.3 ± 1.25^#^
ATG4B (ng/ml)	15.2 ± 2.23	12.7 ± 1.66^∗^	9.1 ± 1.13^#^	3.8 ± 0.84^#^
IL-1*β* (pg/mL)	0.5 ± 0.13	2.2 ± 0.25^∗^	7.2 ± 1.55^#^	11.5 ± 2.43^#^
IL-18 (pg/mL)	6.5 ± 1.29	20.5 ± 3.71^∗^	48.2 ± 4.81^#^	60.1 ± 5.68^#^

Control: healthy people; UACR: urea albumin creatinine ratio; AIF-1: allograft inflammatory factor-1; miR-34a: microRNA 34a; ATG4B: autophagy related 4 homolog B; NLRP3: NOD-like receptor thermal protein domain associated protein 3; IL-1*β*: interleukin 1*β*; IL-18: interleukin 18; Group 1: 30 mg/g ≤ ACR ≤ 300 mg/g; Group 2: ACR > 300 mg/g; Group 3: normal control group; versus control, ^∗^*P* < 0.01, #*P* < 0.001.

**Table 3:** The levels of biochemical indexes and inflammatory factors in *db/db* mice for 4-12 weeks (*n* = 6).

Parameters	*db/m* mice	*db/db* mice		
	4-12 weeks	4 weeks	8 weeks	12 weeks
Blood glucose (mmol/L)	9.3 ± 2.42	17.7 ± 3.61^∗^	24.8 ± 4.33^#^	26.4 ± 5.03^#^
Total cholesterol (mmol/L)	2.5 ± 0.41	3.9 ± 0.85	4.5 ± 1.08^∗^	5.8 ± 1.14^#^
Triglycerides (mmol/L)	1.1 ± 0.12	1.7 ± 0.28	3.4 ± 0.64^∗^	6.2 ± 1.38^#^
Serum creatinine (mg/L)	0.2 ± 0.08	0.3 ± 0.11	0.5 ± 0.17^∗^	1.2 ± 0.25^#^
BUN (mmol/L)	8.7 ± 3.19	9.3 ± 3.76	9.7 ± 4.41	11.4 ± 4.97^∗^
UACR (*μ*g/mg)	32.6 ± 6.15	64.1 ± 10.39^∗^	78.7 ± 12.51^#^	93.5 ± 14.72^#^
AST (unit/L)	21.8 ± 4.76	25.7 ± 5.15	27.1 ± 5.68	25.2 ± 5.47
ALT (unit/L)	41.4 ± 7.11	48.2 ± 6.92	52.4 ± 8.93	51.4 ± 8.17
Total protein (g/dL)	4.3 ± 1.13	3.9 ± 0.97	3.4 ± 0.81^∗^	3.1 ± 0.76^∗^
AIF-1 (pg/mL)	4.3 ± 1.12	12.5 ± 2.34^∗^	25.2 ± 3.54^#^	45.6 ± 5.2^#^
miR-34a (fold)	0.9 ± 0.11	1.3 ± 0.12^∗^	2.1 ± 0.25^#^	3.5 ± 0.37^#^
NLRP3 (ng/ml)	1.2 ± 0.22	2.6 ± 0.32^∗^	5.7 ± 1.62^#^	12.5 ± 2.25^#^
ATG4B (ng/ml)	8.1 ± 1.81	6.7 ± 1.52^∗^	4.5 ± 0.89^#^	1.5 ± 0.32^#^
IL-1*β* (pg/mL)	1.1 ± 0.21	2.3 ± 0.31^∗^	5.8 ± 0.92^#^	9.5 ± 1.83^#^
IL-18 (pg/mL)	5.1 ± 1.15	9.7 ± 2.17^∗^	16.5 ± 3.19^#^	26.5 ± 3.46^#^

BUN: urea nitrogen; UACR: urea albumin creatinine ratio; AST: aspartate aminotransferase; ALT: alanine transaminase; AIF-1: allograft inflammatory factor-1; miR-34a: microRNA 34a; ATG4B: autophagy related 4 homolog B; NLRP3: NOD-like receptor thermal protein domain associated protein 3; IL-1*β*: interleukin 1*β*; IL-18: interleukin 18; versus *db/m* mice, ^∗^*P* < 0.01, #*P* < 0.001.

**Table 4:** The levels of AIF-1, miR-34a, ATG4B, and inflammatory factors in *AIF-1^+/+^* and *AIF-1^−/−^* mice for 12 weeks (*n* = 6).

Parameters	12 weeks		
	Wild mice	*AIF-1^+/+^* mice	*AIF-1^−/−^* mice
Blood glucose (mmol/L)	10.1 ± 4.42	19.4 ± 5.85^∗^	16.7 ± 5.76^∗^
Total cholesterol (mmol/L)	2.8 ± 0.93	4.3 ± 1.26^∗^	4.8 ± 1.58^∗^
Triglycerides (mmol/L)	1.3 ± 0.45	2.4 ± 0.47^∗^	2.1 ± 0.39^∗^
Serum creatinine (mg/L)	0.3 ± 0.17	0.4 ± 0.15	0.6 ± 0.24^∗^
BUN (mmol/L)	6.9 ± 2.75	9.8 ± 3.89^∗^	14.3 ± 6.64^∗^
UACR (*μ*g/mg)	32.6 ± 6.15	64.1 ± 10.39^∗^	78.7 ± 12.51^#^
AST (unit/L)	27.3 ± 5.19	25.2 ± 5.84	22.6 ± 4.16
ALT (unit/L)	46.4 ± 5.12	53.2 ± 5.46	51.6 ± 7.53
Total protein (g/dL)	5.2 ± 1.37	4.6 ± 1.92	3.5 ± 1.47^∗^
AIF-1 (pg/mL)	54.6 ± 4.32	182.7 ± 15.61^∗^	10.3 ± 1.25^#^
miR-34a (fold)	3.9 ± 0.55	6.7 ± 1.82^∗^	1.4 ± 0.13^#^
NLRP3 (ng/ml)	5.5 ± 1.27	9.6 ± 2.14^∗^	1.7 ± 0.21^#^
ATG4B (ng/ml)	1.3 ± 0.32	0.6 ± 0.07^∗^	6.5 ± 1.45^#^
IL-1*β* (pg/mL)	9.5 ± 2.35	22.6 ± 3.42^∗^	3.2 ± 0.84^#^
IL-18 (pg/mL)	15.1 ± 2.87	36.1 ± 3.67^∗^	6.1 ± 1.27^#^

BUN: urea nitrogen; UACR: urea albumin creatinine ratio; AST: aspartate aminotransferase; ALT: alanine transaminase; AIF-1: allograft inflammatory factor-1; miR-34a: microRNA 34a; ATG4B: autophagy related 4 homolog B; NLRP3: NOD-like receptor thermal protein domain associated protein 3; IL-1*β*: interleukin 1*β*; IL-18: interleukin 18; versus wild mice, ^∗^*P* < 0.01, #*P* < 0.001.

**Table 5:** The levels of AIF-1, miR-34a, ATG4B, and inflammatory factors in serum of *db/db* mice treatment with miR-34a agonist or antagonist for 12 weeks (*n* = 6).

Parameters	*db/db* mice (12 weeks)		
	Control	miR-34a agonist	miR-34a antagonist
Blood glucose (mmol/L)	18.7 ± 5.24	20.8 ± 4.76	22.4 ± 6.18
Total cholesterol (mmol/L)	4.2 ± 1.03	4.7 ± 1.57	3.8 ± 1.43
Triglycerides (mmol/L)	2.7 ± 0.88	3.1 ± 1.08	3.5 ± 1.41
Serum creatinine (mg/L)	1.1 ± 0.46	1.4 ± 0.54^∗^	0.8 ± 0.39^∗^
BUN (mmol/L)	11.7 ± 3.17	13.2 ± 4.63	10.6 ± 5.11
UACR (*μ*g/mg)	94.1 ± 16.19	110.6 ± 18.46^#^	43.7 ± 13.82^#^
AST (unit/L)	21.3 ± 4.32	24.6 ± 3.72	22.9 ± 4.48
ALT (unit/L)	42.6 ± 4.81	46.7 ± 4.66	41.2 ± 5.34
Total protein (g/dL)	4.2 ± 1.05	5.5 ± 1.45^∗^	2.5 ± 0.58^#^
AIF-1 (pg/mL)	56.1 ± 6.51	60.1 ± 7.45^∗^	58.6 ± 6.29^#^
miR-34a (fold)	4.1 ± 0.63	11.7 ± 1.86^∗^	0.3 ± 0.12^#^
NLRP3 (ng/ml)	5.3 ± 1.24	19.6 ± 3.76^∗^	2.3 ± 0.54^#^
ATG4B (ng/ml)	1.5 ± 0.41	0.2 ± 0.08^∗^	9.8 ± 2.23^#^
IL-1*β* (pg/mL)	9.1 ± 1.55	38.1 ± 3.27^∗^	1.7 ± 0.26^#^
IL-18 (pg/mL)	13.5 ± 1.98	53.7 ± 4.81^∗^	3.4 ± 0.44^#^

BUN: urea nitrogen; UACR: urea albumin creatinine ratio; AST: aspartate aminotransferase; ALT: alanine transaminase; AIF-1: allograft inflammatory factor-1; miR-34a: microRNA 34a; ATG4B: autophagy related 4 homolog B; NLRP3: NOD-like receptor thermal protein domain associated protein 3; IL-1*β*: interleukin 1*β*; IL-18: interleukin 18; versus control, ^∗^*P* < 0.01, #*P* < 0.001.

## Data Availability

The major data used to support the findings of this study are included within the article and other data available from the corresponding author upon request.
